# A Comparison of Culture- and PCR-Based Methods to Detect Six Major Non-O157 Serogroups of Shiga Toxin-Producing *Escherichia coli* in Cattle Feces

**DOI:** 10.1371/journal.pone.0135446

**Published:** 2015-08-13

**Authors:** Lance W. Noll, Pragathi B. Shridhar, Diana M. Dewsbury, Xiaorong Shi, Natalia Cernicchiaro, David G. Renter, T. G. Nagaraja

**Affiliations:** Department of Diagnostic Medicine/Pathobiology, Kansas State University, Manhattan, Kansas, United States of America; USDA-ARS-ERRC, UNITED STATES

## Abstract

Culture-based methods to detect the six major non-O157 (O26, O45, O103, O111, O121 and O145) Shiga toxin-producing *E*. *coli* (STEC) are not well established. Our objectives of this study were to develop a culture-based method to detect the six non-O157 serogroups in cattle feces and compare the detection with a PCR method. Fecal samples (n = 576) were collected in a feedlot from 24 pens during a 12-week period and enriched in *E*. *coli* broth at 40° C for 6 h. Enriched samples were subjected to immunomagnetic separation, spread-plated onto a selective chromogenic medium, and initially pooled colonies, and subsequently, single colonies were tested by a multiplex PCR targeting six serogroups and four virulence genes, *stx*1, *stx*2, *eae*, and *ehxA* (culture method). Fecal suspensions, before and after enrichment, were also tested by a multiplex PCR targeting six serogroups and four virulence genes (PCR method). There was no difference in the proportions of fecal samples that tested positive (74.3 vs. 77.4%) for one or more of the six serogroups by either culture or the PCR method. However, each method detected one or more of the six serogroups in samples that were negative by the other method. Both culture method and PCR indicated that O26, O45, and O103 were the dominant serogroups. Higher proportions (*P* < 0.05) of fecal samples were positive for O26 (44.4 vs. 22.7%) and O121 (22.9 vs. 2.3%) serogroups by PCR than by the culture method. None of the fecal samples contained more than four serogroups. Only a small proportion of the six serogroups (23/640; 3.6%) isolated carried Shiga toxin genes. The culture method and the PCR method detected all six serogroups in samples negative by the other method, highlighting the importance of subjecting fecal samples to both methods for accurate detection of the six non-O157 STEC in cattle feces.

## Introduction

Cattle are asymptomatic reservoirs for Shiga toxin-producing *Escherichia coli* (STEC), which are major foodborne pathogens [1]. The organisms reside in the hindgut of cattle and are shed in feces, which can serve as a source of contamination of beef, produce, and water for infections in humans [2, 3]. Human illness from an STEC infection can result in clinical manifestations ranging from mild to bloody diarrhea to potentially life-threatening complications, such as hemolytic uremic syndrome primarily in children, but also in other individuals. *Escherichia coli* O157:H7, which was declared an adulterant in 1994 by the USDA Food Safety and Inspection Service (FSIS), has been the focus of many studies, because of the serotype’s predominance in human STEC infections [4, 5]. As a result, detection methods for STEC O157 in cattle feces are well established [6–9]. In recent years, STEC belonging to six O groups, O26, O45, O103, O111, O121, and O145, often referred to as ‘non-O157 STEC’ have been recognized as a growing public health concern [5, 10]. According to the CDC, the six serogroups account for a majority of non-O157 STEC infections in the U.S. [5, 11]. In 2011, the FSIS declared the six serogroups carrying *stx*1 and/or *stx*2 with *eae* genes as adulterants in ground beef and non-intact beef products [12, 13].

Due to the relatively recent recognition of the six non-O157 serogroups, studies on the methodology and standardization of the procedure for detection and isolation, particularly in fecal samples of cattle are limited. PCR-based methods, including commercially available automated methods such as BAX System (DuPont, Wilmington, DE) have been developed for detection of the six non-O157 serogroups and/or virulence genes from cattle feces [14–18]. Although PCR methods are sensitive and are of high-throughput, the inherent limitation is that the presence of virulence genes cannot be associated with any specific serogroups. Also, unlike the culture method, there is no isolate obtained for follow-up analysis and PCR does not discriminate based on viability of cells, which can result in amplification of DNA from dead cells. Despite these advantages over PCR method, development of culture methods for detection of non-O157 STEC has lagged. Identification of O157 STEC has relied on the organism’s inability to ferment sorbitol [19]; however, no such unique phenotypic characteristic is associated with all six non-O157 serogroups, making culture-based identification problematic. A few differential media based on a chromogenic compound to detect β-galactosidase activity and one or more fermentative sugars combined with or without inhibitory compounds (novobiocin, tellurite, etc.) have been developed to detect non-O157 STEC in cattle feces [20–22]. However, differentiation of the six non-O157 serogroups based on colony phenotype is not reliable.

We have utilized an 11-plex PCR [16] assay that targeted the seven serogroups and four major virulence genes (*stx*1, *stx*2, *eae*, and *ehxA*) to determine prevalence of the O157 and six non-O157 serogroups in cattle feces collected from a commercial feedlot [23]. In that study, a culture method that included an immunomagnetic separation (IMS) procedure and plating on a relatively non-selective medium (MacConkey agar) was utilized to determine fecal prevalence of only three serogroups, O26, O103 and O111, because IMS beads for the other three serogroups, O45, O121, and O145, were not available at that time. Some studies have determined the prevalence of non-O157 STEC on beef carcasses [24] and in commercial ground beef [25, 26]. Only a few have investigated fecal shedding of one or more of the non-O157 serogroups in cattle by culture method [18, 21, 22, 27–29]. Our objectives of this study were to develop a culture-based method to detect the six non-O157 (O26, O45, O103, O111, O121, and O145) STEC serogroups in cattle feces and compare the detection of the six serogroups with a PCR method (10). The culture method described here involved an enrichment step, followed by IMS with serogroup-specific beads for each target serogroup, plating on a chromogenic selective medium and confirming the serogroup and major virulence genes by a multiplex PCR. Although PCR detected significantly higher proportions of samples positive for O26 and O121 serogroups compared to the culture method, each method detected all six serogroups in some samples that were negative by the other method, indicating that both methods are required to provide an accurate detection of the presence of non-O157 STEC in cattle feces.

## Materials and Methods

### Animals and fecal sample collection and enrichment

Fecal samples of crossbred finishing cattle were collected from pens in a commercial feedlot in the central US. The permission to collect pen-floor samples was granted by the feedlot Manager and the Nutritionist responsible for feeding and management of the feedyard. The feedlot’s standard operating procedures were followed for care and management of cattle throughout the study period. Kansas State University Institutional Animal Care and Use Committee approved the study (IACUC # 3172). Fecal samples were collected during a 12-week period from June through August 2013. Each week, 24 pen-floor fecal samples were collected from each of two pens that housed an average of 270 cattle per pen. Samples (approximately 100 g) were collected from freshly defecated fecal pats using a plastic spoon, and care was taken to avoid ground contamination. The spoon with feces was placed into a Whirl-pak bag (Nasco, Ft. Atkinson, WI), and once samples were collected, they were placed in a cooler with ice packs and transported to the laboratory in cold storage for processing within 36 h. The sample collection was completed in the late afternoon 12 h prior to transportation of cattle for slaughter. In the laboratory, the sample was mixed by kneading the Whirl-pak bag and approximately 2 g of feces were added to 18 ml of *Escherichia coli* broth (EC medium; Difco, Becton, Dickinson Co., Sparks, MD), vortexed for 30 s and incubated at 40°C for 6 h [17].

### Culture-based detection and isolation

Enriched fecal suspensions were subjected to IMS procedure and plated onto a selective medium to detect and isolate O26, O45, O103, O111, O121, and O145 serogroups of *E*. *coli*. Each fecal sample was individually subjected to six serogroup-specific IMS beads. Nine-hundred and eighty microliters of enriched sample were mixed with 20 μl of serogroup-specific IMS beads (Abraxis, Warminster, PA) in 96-well microtiter plates. The IMS procedure was carried out in a Kingfisher Flex Magnetic Particle Processor (Thermo Scientific, Waltham, MA) according to the protocol provided by the manufacturer. Then, 50 μl of the sample bead suspensions of the non-O157 serogroups were spread-plated onto Possé agar medium [20] modified to include novobiocin at 5 mg/l and potassium tellurite at 0.5 mg/l. Plates were incubated for 20 to 24 h at 37°C. Six chromogenic colonies (mauve, green, blue or purple) from the modified Possé medium were picked, inoculated onto blood agar (Remel, Lenexa, KS) plates, and incubated at 37°C for 24 h. Six colonies from each sample were pooled in distilled water, boiled for 10 min, centrifuged at 9,300 x g for 5 min, and the lysate containing the DNA was tested by a multiplex PCR assay that targeted genes specific to the six serogroups (*wzx* gene for O26, O45, O103, O111, and O145 and *wbqE* and *wbq* for O121). Primers for the O26, O103, O121, and O145, serogroups were according to Bai et al. [16], while the O45 serogroup primers were according to Paddock et al. [17]. The primers for the *wzx* gene of O111 were modified from Bai et al. [16] and sequences were as follows: O111-F3, ACA AGA GTG CTC TGG GCT TC and O111-R3, AAA CTA AGT GAG ACG CCA CCA. If the pooled colonies were positive for any of the six non-O157 serogroups, then each of the six isolates were tested by a ten-plex PCR targeting six serogroups and four virulence genes (*stx*1, *stx*2, *eae*, and *ehxA*) [16]. The primers for the four virulence genes were according Bai et al. [16], except for *eae*, which were changed to: *eae*-F2, TAC GCG AAA GAT ACC GCT CT and *eae*-R2, CAT GCG GAA ATA GCC GTT A. Colonies isolated from any serogroup-specific IMS beads that tested positive for a different serogroup were considered positive by non-serogroup specific IMS beads. If pooled colonies were PCR negative for any of the six serogroups, the sample was considered negative. Isolates confirmed to be positive for one of the six serogroups were stored in cryogenic beads (CryoCare, Key Scientific Products, Round Rock, TX).

### PCR-based detection

One milliliter aliquots of the fecal suspension in EC broth before (pre-enrichment) and after incubation (post-enrichment) were boiled for 10 min, then centrifuged at 9,300 x g for 5 min. One hundred μl of crude DNA, from pre- and post-enriched samples, were then purified with GeneClean® Turbo Kits (MP Biomedicals, Solon, OH) and subjected to a multiplex PCR assay [16] to detect six O serogroups (O26, O45, O103, O111, O121, and O145) and four virulence genes (*stx*1, *stx*2, *eae* and *ehxA*).

### Data analysis

Fecal samples were considered positive for one or more of the six non-O157 serogroups if the enriched sample, subjected to IMS and plated on modified Possé medium, tested positive by the multiplex PCR of the pooled colonies (culture method) or by the direct multiplex PCR (PCR method) of the enriched fecal sample. Isolates of serogroups from the culture method testing positive for Shiga toxin genes (*stx*1 and/or *stx*2) were considered STEC. The proportion of fecal samples tested positive by culture- or PCR-based method was computed by dividing the number of samples testing positive for each O serogroup by the total number of samples tested (n = 576). A two-sample test of proportions was performed to determine whether the proportions of positive samples differed significantly between the two detection methods. In addition, the proportion of samples that tested positive for the three predominant serogroups, O26, O45, and O103, detected by culture only, PCR only, culture or PCR, and culture and PCR methods were compared. Furthermore, to assess overall agreement between the two methods for detection of O26, O45, and O103 serogroups, the Cohen’s Kappa statistic (and 95% confidence intervals) and McNemar’s chi-square test were calculated using STATA MP 11.0 (kap and mcc commands, StataCorp, College Station, TX) Interpretations of the kappa statistic were based on the scale proposed by Landis and Koch [30].

## Results

### Culture method of detection of six non-O157 serogroups

A total of 428 samples (74.3%) tested positive for one or more of the six serogroups of *E*. *coli*. Fecal samples were identified as positive for non-O157 serogroups, if pooled colonies (up to six per sample) from either serogroup-specific or non-serogroup specific IMS beads were positive by the multiplex PCR (Table 1). Based on serogroup-specific IMS beads, O103 was detected in 37.5% of the samples (216/576), followed by O26 (17.0%), O45 (15.6%), O145 (2.1%), O121 (1.7%), and O111 (0.2%). The proportion positive for each serogroup, except O111, which was present in only one fecal sample, increased when samples that tested positive for those serogroups from non-serogroup specific IMS beads were included (Table 1). The O103 and O26 beads were less specific as they identified only 62.2% (216/347) and 74.8% (98/131) of the total samples positive for their target O serogroups, respectively. The most common serogroup identified using non-serogroup specific IMS beads was O103. Nearly 38% (131/347) of all O103 positive samples were detected by non-O103 IMS beads, but were tested as negative with the O103 bead. The O26 serogroup was identified by non-O26 beads in 25.2% (33/131) of all O26 positive samples. Only 6.3% (6/96) of all O45-positive samples were identified by non-O45 IMS beads. The O145 and O121 serogroups were identified by non-target beads in 29.4% (5/17) and 23.1% (3/13) of positive samples, respectively.

**Table 1:** Detection of six serogroups of non-O157 *Escherichia coli* in feces (576) of feedlot cattle by culture-based method involving enrichment, immunomagnetic bead separation (IMS) and plating on a selective medium.

IMS beads	Number of samples positive (%)^†^ ^,^ ^‡^					
	O26	O45	O103	O111	O121	O145
**O26**	**98 (17.0)**	5	128	0	0	3
**O45**	18	**90 (15.6)**	118	0	0	0
**O103**	20	4	**216 (37.5)**	0	1	2
**O111**	22	9	99	**1 (0.2)**	1	1
**O121**	22	4	117	0	**10 (1.7)**	3
**O145**	12	7	110	0	2	**12 (2.1)**
	* **Total non-redundant positives**					
	**131 (22.7)**	**96 (16.7)**	**347 (60.2)**	**1 (0.2)**	**13 (2.3)**	**17 (3.0)**

^†^Feces were enriched in *Escherichia coli* broth for 6 h at 40°C.

^‡^Enriched samples, subjected to serogroup-specific IMS beads, were plated onto modified Possé medium [20] modified to include novobiocin at 5 mg/l and potassium tellurite at 0.5 mg/l and then up to six chromogenic colonies were pooled and tested by a multiplex PCR assay [17] targeting serogroup-specific genes.

*Non-redundant positive data include samples that were positive for a serogroup by serogroup-specific and non-specific IMS beads.

After accounting for redundancy in samples that were identified as positive for a serogroup by multiple IMS beads, the O103 was the most predominant serogroup with 60.2% (347/576) of samples testing positive. The O26 and O45 serogroups were detected in 22.7% (131/576) and 16.7% (96/576) of samples, respectively. The other three serogroups, O145 (3.0%), O121 (2.3%) and O111 (0.2%), were detected in lower proportions. The proportion of multiple serogroups detected within a fecal sample by the culture-based method is shown in Table 2. None of the fecal samples tested positive for five or six serogroups and the majority (50.7%) tested positive for a single serogroup.

**Table 2:** Number and percentage of samples testing positive to multiple non-O157 *Escherichia coli* serogroups in fecal samples (n = 576) of feedlot cattle based on culture method and multiplex PCR method of detection.

Detection method		Number of serogroups (%)						
	Total positive	0	1	2	3	4	5	6
**Culture-based**	428 (74.3)	148 (25.7)	292 (50.7)	100 (17.4)	31 (5.4)	5 (0.9)	0	0
**PCR-based**	446 (77.4)	130 (22.6)	180 (31.3)	170 (29.5)	72 (12.5)	24 (4.2)	0	0

### Major virulence genes in isolates of six non-O157 serogroups

A total of 640 non-O157 isolates were obtained from 576 fecal samples in the study (Table 3). Isolates with identical virulence gene profiles, recovered from the same fecal sample from one or more IMS beads, were recorded once. Isolates with differing virulence gene profiles from the same fecal sample, were recorded separately, regardless from which IMS bead they were recovered. All O121 (n = 12) and O111 (n = 1) isolates tested negative for *stx*1, *stx*2, *eae* and *ehxA* genes. Only small proportions of O26 (7.6%; 10/132) and O145 (5.3%; 1/19) isolates were negative for all four virulence genes (Table 3). Conversely, high proportions of O103 and O45 were negative for all four virulence genes (79.8% [304/381] and 58.9% [56/95] of O103 and O45, respectively). A total of 23 *stx-*positive isolates were obtained, which included serogroups O103 (10), O26 (7), and O145 (6) (Table 3). The *stx*1 gene was detected in 20 of the 23 (87.0%) isolates, and *stx*2 was identified only in isolates of serogroups O103 (1) and O145 (2). None of the non-O157 STEC isolates contained both *stx* genes and all were positive for *eae*. High proportions of O26 (122/132; 92.4%) and O145 (17/19; 89.5%) isolates tested positive for *eae* compared with 19.7% (75/381) for O103 isolates. None of the O45 isolates (n = 95) tested positive for *eae*. A high proportion of O145 isolates (89.5%) tested positive for the *ehxA* gene compared with O26, (9.8%), O45 (41.1%) and O103 (19.9%) isolates (Table 3). A total of 99 *stx*-positive isolates were recovered that were not associated with any of the six non-O157 or O157 serogroups, and only a small proportion of those (22.2%) tested positive for *eae* (data not shown).

**Table 3:** Distribution of major virulence genes in six non-O157 serogroups of *Escherichia coli* isolated from fecal samples (n = 576) of feedlot cattle^†^.

Virulence genes (*stx*1, *stx*2, *eae*, *ehxA*)	Number of six serogroups of non–O157 isolates (%)						
	O26 (n = 132)	O45 (n = 95)	O103 (n = 381)	O111 (n = 1)	O121 (n = 12)	O145 (n = 19)	Total (n = 640)
**None**	10 (7.6)	56 (58.9)	304 (79.8)	1 (100)	12 (100)	1 (5.3)	384 (60.0)
***stx*1**	7 (5.3)	0	9 (2.4)	0	0	4 (21.1)	20 (3.1)
***stx*2**	0	0	1 (0.3)	0	0	2 (10.5)	3 (0.5)
***eae***	122 (92.4)	0	75 (19.7)	0	0	17 (89.5)	214 (33.4)
***ehxA***	13 (9.8)	39 (41.1)	76 (19.9)	0	0	17 (89.5)	145 (22.7)
***stx*1 +*stx*2**	0	0	0	0	0	0	0
***stx*1 + *eae***	7 (5.3)	0	9 (2.4)	0	0	4 (21.1)	20 (3.1)
***stx*2 + *eae***	0	0	1 (0.3)	0	0	2 (10.5)	3 (0.5)
***stx*1 or *stx*2 + *eae***	7 (5.3)	0	10 (2.6)	0	0	6 (31.6)	23 (3.6)

^**†**^Feces were enriched in *Escherichia coli* broth for 6 hours at 40°C. Enriched samples were subjected to serogroup-specific immunomagnetic separation, plated onto modified Possé medium [20] and then up to six chromogenic colonies were pooled and tested by a multiplex PCR assay targeting serogroup-specific genes [17]. If pooled colonies tested positive for any of the target serogroups, individual colonies were tested by PCR for confirmation of serogroup and identification of four virulence genes [16].

### PCR detection of six non-O157 serogroups and four virulence genes

In pre-enriched samples (n = 576), a total of 90 samples (15.6%) tested positive for one or more of the six serogroups, with O103 and O45 the most commonly detected serogroups (5.9 and 5.2%, respectively; Table 4) by the direct PCR method. None of the pre-enriched samples were positive for the O145 serogroup. In post-enriched samples, a total of 446 samples (77.4%) tested positive for one or more of the six serogroups. The O103 serogroup was the most frequently identified serogroup, with 56.6% of the samples (326/576) testing positive. The O26 serogroup (44.4%) was the second most predominant, followed by O121 (22.9%), and O45 (17.9%). Serogroups O111 and O145 were detected in only 0.7% and 1.9% of samples, respectively. Among the four virulence genes, *stx*2, *eae*, and *ehxA* were detected in a high proportion (94.1 to 99.5%) of enriched samples (Table 4). A significantly greater (*P* < 0.05) proportion of fecal samples were positive for *stx*2 (94.1%) than *stx*1 (64.4%). Interestingly, before enrichment, a significantly greater (*P* < 0.05) proportion of samples tested positive for *stx*1 (35.9%) than for *stx*2 (23.1%). Before enrichment, a total of 270 (46.9%) samples tested positive for at least one *stx* gene and 83 (30.7%) of those samples tested negative for any of the six non-O157 serogroups. After enrichment, a total of 552 (95.8%) samples tested positive for at least one *stx* gene, and 193 (35.0%) of those samples tested negative for any of the six non-O157 serogroups. The proportion of fecal samples testing positive for multiple serogroups by PCR is shown in Table 2. A high proportion of samples (73.2%) tested positive for up to three serogroups. None of the fecal samples tested positive for five or six serogroups.

**Table 4:** Detection of six major serogroups and virulence genes of non-O157 Shiga toxin-producing *Escherichia coli*, based on multiplex PCR assay, in fecal samples (n = 576) of feedlot cattle.

Enrichment^†^		Serogroup-specific genes, no. positive (%)						Virulence genes, no. positive (%)			
	Total positives^‡^	O26	O45	O103	O111	O121	O145	*stx*1	*stx*2	*eae*	*ehxA*
**Before**	90 (15.6)	19 (3.3)	30 (5.2)	34 (5.9)	2 (0.3)	21 (3.6)	0 (0)	207 (35.9)	133 (23.1)	122 (21.2)	327 (56.8)
**After**	446 (77.4)	256 (44.4)	103 (17.9)	326 (56.6)	4 (0.7)	132 (22.9)	11 (1.9)	371 (64.4)	542 (94.1)	561 (97.4)	573 (99.5)

^†^Feces were enriched in *Escherichia coli* broth for 6 h at 40°C.

^‡^Samples positive for one or more of the six serogroups of *E*. *coli*.

### Culture vs. PCR methods for the detection of six non-O157 serogroups

**Fig 1:**
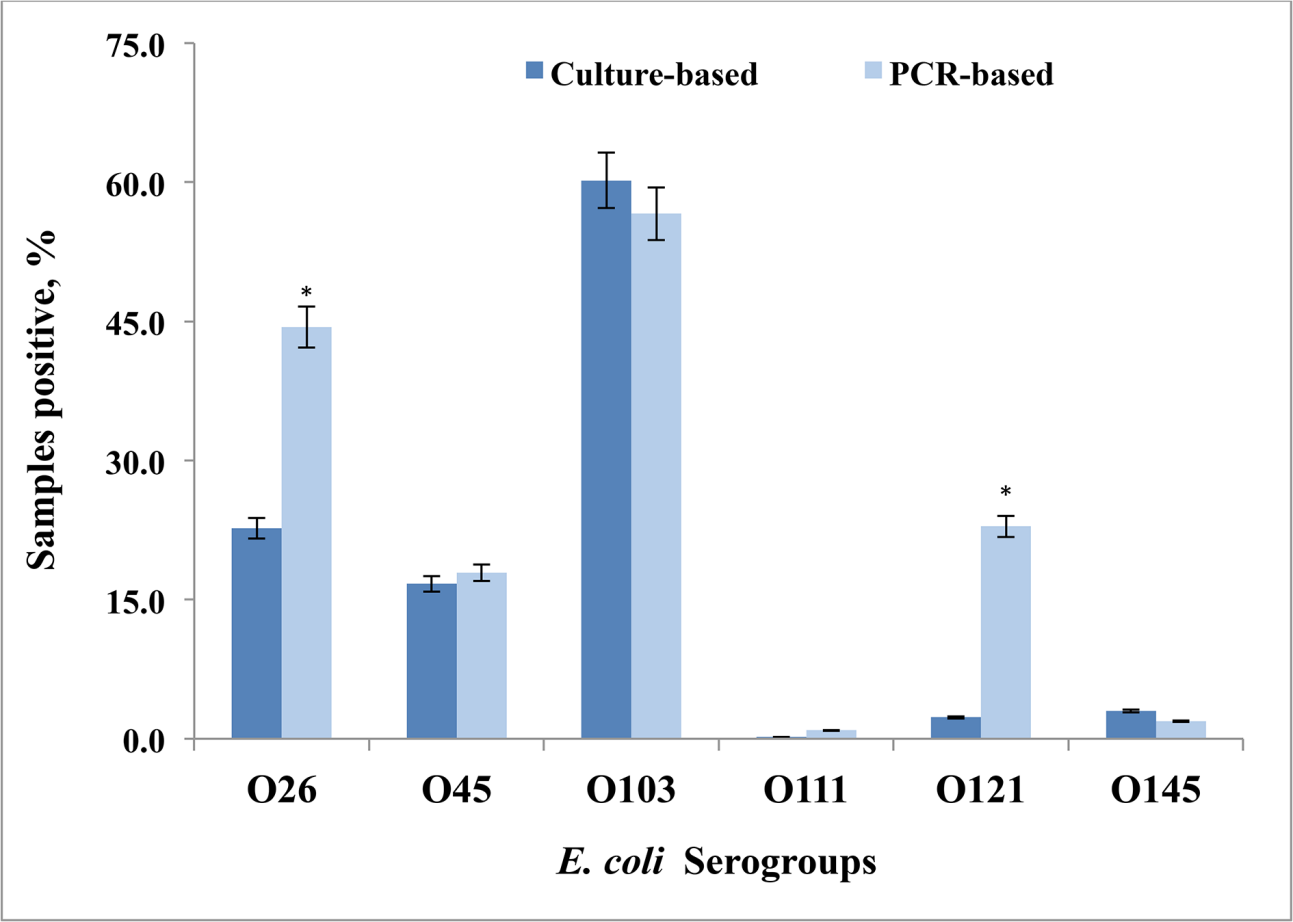
Detection of the six major non-O157 serogroups of *Escherichia coli*, based on a culture- or multiplex PCR-based method, in fecal samples of feedlot cattle (n = 576). *Denotes significant difference in proportions within serogroups (*P* < 0.05).

The culture and PCR methods did not differ in the proportions of fecal samples that tested positive (74.3 vs. 77.4%) or negative (25.7 and 22.6%) for one or more of the six non-O157 serogroups (Table 2). A higher proportion of samples were positive for O26, O45, O111, and O121 serogroups by PCR than by the culture method (Fig 1); however, only differences in the proportions of O26 and O121 serogroups were statistically significant (*P* < 0.001) between the two methods. The O103 and O145 serogroups were detected slightly more often with the culture method than the PCR method, but these proportions were not significantly different. A total of 266 (46.2%) samples tested positive after enrichment for more than one serogroup (2 to 4 serogroups) by PCR compared with only 136 (23.6%) of samples by the culture method (Table 2). The PCR assay detected a higher proportion of fecal samples as positive for three or four serogroups than the culture method (16.7 and 6.3%, respectively; *P* < 0.05) and the culture method detected a higher percentage (*P* < 0.05) of samples positive for 1 or 2 serogroups (68.1%) compared with the PCR based method (60.8%; Table 2). Samples testing positive for a single serogroup by both PCR and culture-based method were further analyzed for predominance of a particular serogroup. For the PCR-based method, 35.5% (50/141) of samples that tested positive for a single serogroup were positive for the O103 serogroup. The culture-based method detected the O103 serogroup in 54.6% (119/218) of samples that were positive for a single serogroup. Samples positive for a single serogroup other than O103 were detected at similar levels with both detection methods.

Because high proportions of fecal samples were positive for O26, O45, and O103 serogroups, detection capabilities by either or both methods were compared (Table 5). A significantly (*P* < 0.05) higher proportion of samples tested positive for O26 and O121 serogroups by PCR only than by the culture-based detection only. Samples positive for the three serogroups were detected by both methods in only 28.6% (86/301), 30.1% (46/153), and 56.5% (243/430) for O26, O45, and O103 serogroups, respectively (Table 5). The PCR method detected O26 (56.5%), O45 (37.3%), and O103 (19.3%) in samples that were negative by the culture method. Conversely, the culture method detected O26 (15.0%), O45 (32.7%), and O103 (24.2%) in samples that were negative by the PCR method. There was a slight to fair agreement (Kappa range: 0.01–0.4) between the PCR only and culture-based only methods for detection of O26, O45, and O103 serogroups in fecal samples (Table 6); however, the McNemar’s Chi-square tests for the comparison between tests for detection of the O26 serogroup was statistically significant (*P* < 0.001), indicating a disagreement between the proportions of positive samples detected by the two methods.

**Table 5:** Detection of *Escherichia coli* O26, O45, and O103 serogroups, based on culture method and/or multiplex PCR method of detection, in fecal samples (n = 576) of feedlot cattle.

Serogroups			Number positive of the total samples positive (%)			
	Total negatives^†^ (n = 576)	Total positives^‡^ (n = 576)	Culture only	PCR only	Culture or PCR	Culture and PCR
**O26**	275 (47.7)	301 (52.3)	45 (15.0)^a^	170 (56.5)^b^	215 (71.4)^c^	86 (28.6)^d^
**O45**	423 (73.4)	153 (26.6)	50 (32.7)^a^	57 (37.3)^a^	107 (69.9)^b^	46 (30.1)^a^
**O103**	146 (25.3)	430 (74.7)	104 (24.2)^a^	83 (19.3)^a^	187 (43.5)^b^	243 (56.5)^c^

^†^ Total negatives include samples negative by culture- and/or PCR-based methods.

^‡^ Total positives include samples positive by culture- and/or PCR-based methods.

^a,b,c,d^ Numbers within the same row with different superscripts are statistically different (*P* < 0.001).

**Table 6:** Agreement between culture and PCR methods for detection of *Escherichia coli* O26, O45, and O103 serogroups in fecal samples of feedlot cattle (n = 576).

Serogroup		Comparison of detection methods				
	Statistics	Culture only vs. PCR only	Culture only vs. PCR and culture	PCR only vs. PCR and culture	Culture only vs. PCR or culture	PCR only vs. PCR or culture
**O26**	Kappa	-0.14	-0.11	-0.25	0.25	0.83
	(95% CI)	(-0.18 –-0.10)	(-0.15 –-0.08)	(-0.30 –-0.20)	(0.19–0.31)	(0.78–0.87)
	McNemar’s χ^2^ (*P*-value)	72.7 (< 0.001)	12.8 (< 0.001)	27.6 (< 0.001)	170.0 (< 0.001)	45.0 (< 0.001)
**O45**	Kappa	-0.10	-0.09	-0.10	0.59	0.65
	(95% CI)	(-0.13 –-0.07)	(-0.12 –-0.06)	(-0.13 –-0.07)	(0.50–0.68)	(0.56–0.74)
	McNemar’s χ^2^ (*P*-value)	0.5 (0.5)	0.2 (0.7)	1.2 (0.3)	57.0 (< 0.001)	50.0 (< 0.001)
						
**O103**	Kappa	-0.19	-0.34	-0.27	0.63	0.52
	(95% CI)	(-0.23 –-0.15)	(-0.40 –-0.28)	(-0.33 –-0.22)	(0.56–0.70)	(0.45–0.59)
	McNemar’s χ^2^ (*P*-value)	2.4 (0.12)	55.7 (< 0.001)	78.5 (< 0.001)	83.0 (< 0.001)	104.0 (< 0.001)

## Discussion

Although the fecal shedding of the O157 serogroup has been studied extensively, only a few studies have examined fecal shedding of non-O157 STEC in cattle, particularly in the United States [18, 22, 23, 29, 31]. This may be due in part to the lack of a validated culture method to detect and isolate non-O157 STEC from cattle feces. The six non-O157 serogroups do not possess unique phenotypic markers (similar to non-sorbitol fermentation by O157) that allow differentiation from other *E*. *coli*, making detection of non-O157 STEC problematic. Some studies have relied on PCR to determine the prevalence of the six serogroups and virulence genes [18, 23, 31]. The inherent limitation of a PCR method is the inability to link virulence genes to target serogroups present in samples. Therefore, we utilized both PCR and culture methods to detect the six serogroups of non-O157 STEC in fecal samples. The culture method included IMS with serogroup-specific beads and plating the bead suspension onto a chromogenic Possé medium [20]. The Possé medium is based on lactose-free MacConkey medium with a mixture of sugars (sucrose and sorbose), β-glucosidase activity and selective components (novobiocin and potassium tellurite), which allows color-based identification (blue to purple to mauve to green) of serogroups. The modification of the medium included lowering the concentration of novobiocin (from 8 to 5 mg/L) and potassium tellurite (from 2.5 to 0.5 mg/L) because certain strains of non-O157 serogroups in our culture collection did not grow on the medium with the original concentrations of these inhibitory compounds [29]. Although the differentiation of serogroups is supposedly based on colony color, distinguishing the phenotypes was difficult because of the shades of colors observed, particularly when the plate was crowded with colonies. Little association between the colors of the colonies and the target non-O157 serogroup was evident. Therefore, we chose to pick multiple “*E*. *coli-*like” colonies (six colonies per plate) with any shades of blue/purple/mauve/green colors. The pooling of colonies and testing by a multiplex PCR allowed us to identify samples that were positive for any of the six serogroups. Subsequent testing of individual colonies permitted us to confirm the samples positive for any of the six serogroups and the virulence genes and to obtain pure cultures of the non-O157 serogroups.

Based on the culture method, O103 was the most commonly detected serogroup; however, the O103 O group was the most common non-specific target of the IMS beads with nearly 38% of O103-positive samples identified using non-O103 IMS beads. The high affinity of non-O103 IMS beads toward the O103 O group could have overestimated the relative proportion of samples that were positive for the O103 serogroup, but PCR-based detection also identified O103 as the predominant serogroup. Both PCR and culture-based detection methods identified the O26 serogroup as the second most common serogroup. However, PCR identified the O26 and O121 serogroups in more samples than the culture-method, likely because PCR detects nonviable cells. Interestingly, Paddock et al. [29] reported detection of the O26 serogroup in cattle feces more often by the culture-based method (22.7%) compared with PCR (10.5%), highlighting the overall lack of agreement between the two detection methods. Both detection methods agreed in identifying the O45 serogroup. In addition, the O45 IMS beads exhibited the most specificity (93.8% of the samples positive for O45) towards the target serogroup, not considering the single O111 serogroup detected by the O111 IMS beads. Other studies have reported difficulty in isolating the O111 serogroup after IMS, possibly owing to a low affinity between the O111 IMS bead antibody and the O111 antigen [32, 33], but in our study, the O111 serogroup-specific gene also was detected in a low proportion of samples by the PCR assay. Other studies have had difficulty recovering certain serogroups from feces [32] and ground beef [33] after IMS. The degree of non-specificity may depend on the source of IMS beads. Cernicchiaro et al. [23] reported a degree of cross-reactivity with Dynabeads (Invitrogen, Carlsbad, CA), suggesting non-specific binding to the IMS beads. Our results confirm the non-specificity of certain IMS beads, possibly contributing to differences in identification of certain serogroups observed between detection methods.

Based on the culture method, only 4.0% (23/576) of fecal samples tested were positive for at least one of the six non-O157 serogroups carrying the *stx* gene, with a higher proportion harboring the *stx*1 gene. Interestingly, Paddock et al. [29] reported a higher proportion of pooled colonies that tested positive for the six non-O157 isolates recovered from cattle feces testing positive for *stx*2 rather than *stx*1. Cernicchiaro et al. [23] reported similar findings, although a higher proportion of O26 isolates were positive for the *stx*1 gene. Interestingly, 31.6% (6/19) of the total O145 isolates identified in the study were STEC. Conversely, the O103 and O26 STEC isolates contributed to only 2.6% (10/381) and 5.3% (7/132) of the total isolates identified for these serogroups, respectively. Numerous non-O157 isolates that tested negative for Shiga toxin genes were isolated in the current study, and the majority (60%) of these isolates were also negative for the *eae* and *ehxA* genes. Nearly 80% (304/381) of all O103 isolates identified were negative for the four virulence genes. Hence, although the O103 serogroup is present in cattle feces at a relatively high proportion, only a small percentage of isolates contained any of the four virulence genes. Conversely, 92.4% (122/134) of O26 isolates and 89.5% (17/19) of O145 isolates were positive for the *eae* gene, suggesting that a majority of these isolates in the cattle feces are capable of intimin-based attachment. Unlike the non-O157 STEC that tested mostly positive for *stx*1, 80.8% (80/99) of the isolates that were not one of the six non-O157 (or O157) serogroups were positive for *stx*2. Considering the low prevalence of non-O157 STEC isolates belonging to the six serogroups, it is possible that serogroups from other non-O157 STEC are more predominant in the cattle feces of this study population. Although, numerous isolates belonged to the O103, O26 and O45 serogroups, the majority did not carry Shiga toxin genes. However, if STEC from the six serogroups as well as from undetermined serogroups (not including O157 serogroup) were included, 18.8% (108/576) of samples tested in the study were positive for STEC. In conclusion, serogroups O103 and O26 were the predominant serogroups in feces of cattle sampled in this study, and PCR detected higher proportions of fecal samples as positive for O26 but not O103 than the culture method. Only a small proportion of the non-O157 serogroup isolates carried the Shiga toxin gene. More importantly, each method (culture and PCR) detected the six non-O157 serogroups in fecal samples that were negative by the other method. This is an important observation because the FSIS method [13] for detecting non-O157 STEC in beef samples is based on PCR detection of *stx* and *eae* genes first, followed by detection of serogroups, and a sample positive for both is then subjected to culture method. Therefore, it will be of interest to compare the culture method and PCR method to detect the six non-O157 serogroups in beef samples. Our data on fecal sample analysis suggest that a sample should be subjected to both culture and PCR method to get an accurate estimate of the presence of the six non-O157 STEC.
